# Light and sound hypersensitivity in autism spectrum disorder: a systematic review focusing on age and gender bias

**DOI:** 10.3389/fpsyt.2026.1771956

**Published:** 2026-02-20

**Authors:** Carlos Ríos Llamas, Diego Oswaldo Camacho Vega, María Guadalupe Delgadillo Ramos

**Affiliations:** 1 Faculty of Architecture and Design, Autonomous University of Baja California, Mexicali, Baja California, Mexico; 2 Faculty of Medicine and Psychology, Autonomous University of Baja California, Tijuana, Baja California, Mexico

**Keywords:** age bias, autism spectrum disorder, gender bias, hypersensitivity, lighting, noise, sensory

## Abstract

**Background:**

Autism spectrum disorder (ASD) is often characterized by hypersensitivity to sensory stimuli, with 70–90% of autistic individuals experiencing such difficulties. In environments without appropriate accommodation, these sensitivities can cause discomfort; however, current guidelines for regulating light and sound remain limited, and evidence from current research indicates a striking asymmetry across ages and genders.

**Objectives:**

This systematic review synthesized 29 studies (2015–2025) to (1): characterize distinct auditory and visual sensory hypersensitivity profiles in ASD compared to typically development (TD) controls (2); identify mechanisms of reduced habituation and impaired multisensory integration (3); quantify and critique methodological biases (age, gender, and context); and (4) map critical research gaps for inclusive environmental studies.

**Methods:**

PRISMA guidelines and registered protocol (PROSPERO: CRD420251042397). Three databases (PubMed, Web of Science, Scopus) resulted in 410 records; 29 met the strict PICO criteria, involving experimental/observational assessment of auditory or visual responses with ASD-TD comparisons. Quality assessment utilized a 16-item DISCERN instrument specifically adapted for neuroscience research. Data extraction encompassed demographic attributes (age, gender) and behavioral outcomes.

**Results:**

Most studies examined auditory stimuli, with few focusing on lighting effects. Sensory responses of autistic individuals were atypical, including reduced habituation to sounds, impaired sensory gating, and increased sensitivity to noises. A natural soundscape is more effective at regulating the body than clinical white noise. Visual research is limited, with no studies on photophobia or modern lighting. Audiovisual studies show delayed speech processing, slower adaptation to asynchrony, and reduced susceptibility to multisensory illusions, reflecting detail-focused perception. Males and children predominate in most studies, and females are underrepresented, particularly in adult studies, creating an age and gender gap.

**Conclusion:**

Addressing critical gaps and biases in autism sensory processing and therapeutic opportunities is essential. Studies should encompass gender-balanced perspectives, lifespan development, and visual hypersensitivity, incorporating ecological validity and translating findings into actionable environmental standards. Rather than perpetuating historical biases, it is crucial to prioritize the needs of underrepresented groups.

**Systematic Review Registration:**

https://www.crd.york.ac.uk/PROSPERO/view/CRD420251042397, identifier CRD420251042397.

## 1 Introduction

Autism spectrum disorder (ASD) is a neurodevelopmental condition characterized by social communication challenges, repetitive behaviors, and atypical sensory processing. Approximately 70–90% of autistic individuals experience heightened sensory sensitivities, often leading to over-responsiveness to everyday stimuli (1–3). These sensory challenges can increase stress and anxiety, negatively affecting mental health and well-being (4). While some sensory sensitivities may decrease with age, masking or camouflaging discomfort can hide outward distress but lead to internalized stress (5).

Studies of ASD focus on the relationship between multisensory temporal function and behavioral, perceptual, and cognitive impairments. In many studies on hypersensitivity in ASD, family members and caregivers are interviewed, but very few experimental and structured studies compare hypersensitivity to neurotypical profiles, acoustics, and lighting conditions. In spite of the large number of hypersensitivity studies that rely on interviews or caregiver reports, controlled experiments are rare, which hinders our understanding of sensory hypersensitivity mechanisms.

Research on ASD primarily examines the correlation between multisensory temporal function and behavioral, perceptual, and cognitive impairments. While numerous studies on hypersensitivity in ASD involve interviews with family members and caregivers, few experimental and structured studies compare hypersensitivity to neurotypical profiles, acoustics, and lighting conditions. Despite the abundance of hypersensitivity studies that rely on interviews or caregiver reports, controlled experiments are scarce, which impedes our comprehension of sensory hypersensitivity mechanisms.

In ASDs, sensory hyper-reactivity is often considered excessive by neurotypical standards. In autistic individuals, certain colors and brightness levels of light, loud or sudden sounds, and certain pitches or background noises can produce strong negative reactions (6). Eventually, some autistic children and adults may appear to be less reactive to the outside world as a result of learned coping techniques. As autistic children grow, social expectations require many to hide and suppress their discomfort. This is a phenomenon that differs with age and is often distinct between men and women (6–8). Research indicates discernible differences in sensory processing between autistic males and females (9). This gender-based disparity, in conjunction with developmental variations, necessitates sensory research to account for individual differences.

Based on the hypothesis that hypersensitivity in individuals with ASD can influence their responses to socialization demands (7, 8), this study aims to integrate existing research to enhance our understanding of how sensory stimuli affect individuals with autism, highlighting areas that warrant further investigation. Specifically, it explores the effects of lighting and noise on sensory hypersensitivity across different age groups and genders.

## 2 Methods

### 2.1 Study design

This systematic review adhered to the Preferred Reporting Items for Systematic Reviews and Meta-Analyses (PRISMA) guidelines. The protocol was registered and approved in the PROSPERO database (CRD420251042397).

### 2.2 Information sources and search strategy

A comprehensive search was conducted on PubMed, Web of Science, and Scopus using the Boolean search strategy: (Autism [Title]) AND (Acoustic Stimulation OR Lighting) AND Sensory. The search was restricted to peer-reviewed articles published between 2015 and 2025, encompassing research on autism, sensory stimulation, lighting, and acoustic hypersensitivity. This 10-year interval was chosen to capture contemporary research in sensory neuroscience and multisensory integration, which has expanded significantly in recent years (10) Safar et al., 2024). Initial results included: PubMed (155 records), Web of Science (103 records), and Scopus (152 records).

### 2.3 Eligibility criteria

The inclusion criteria specified that studies must address sensory processing and environmental modulation in individuals with ASD, employing experimental or observational methods. Additionally, the papers were required to focus on sensory responses to lighting and acoustics, with hypersensitivity evaluated through sensory stimulation interventions (audio, visual, or audiovisual). Baseline sensory responses were determined by pre-post assessments or TD controls. To reduce the initial 410 records to relevant data, duplicates, clinical studies, systematic reviews, abstracts, and letters to the editor were excluded.

### 2.4 Screening procedure

**Figure 1:**
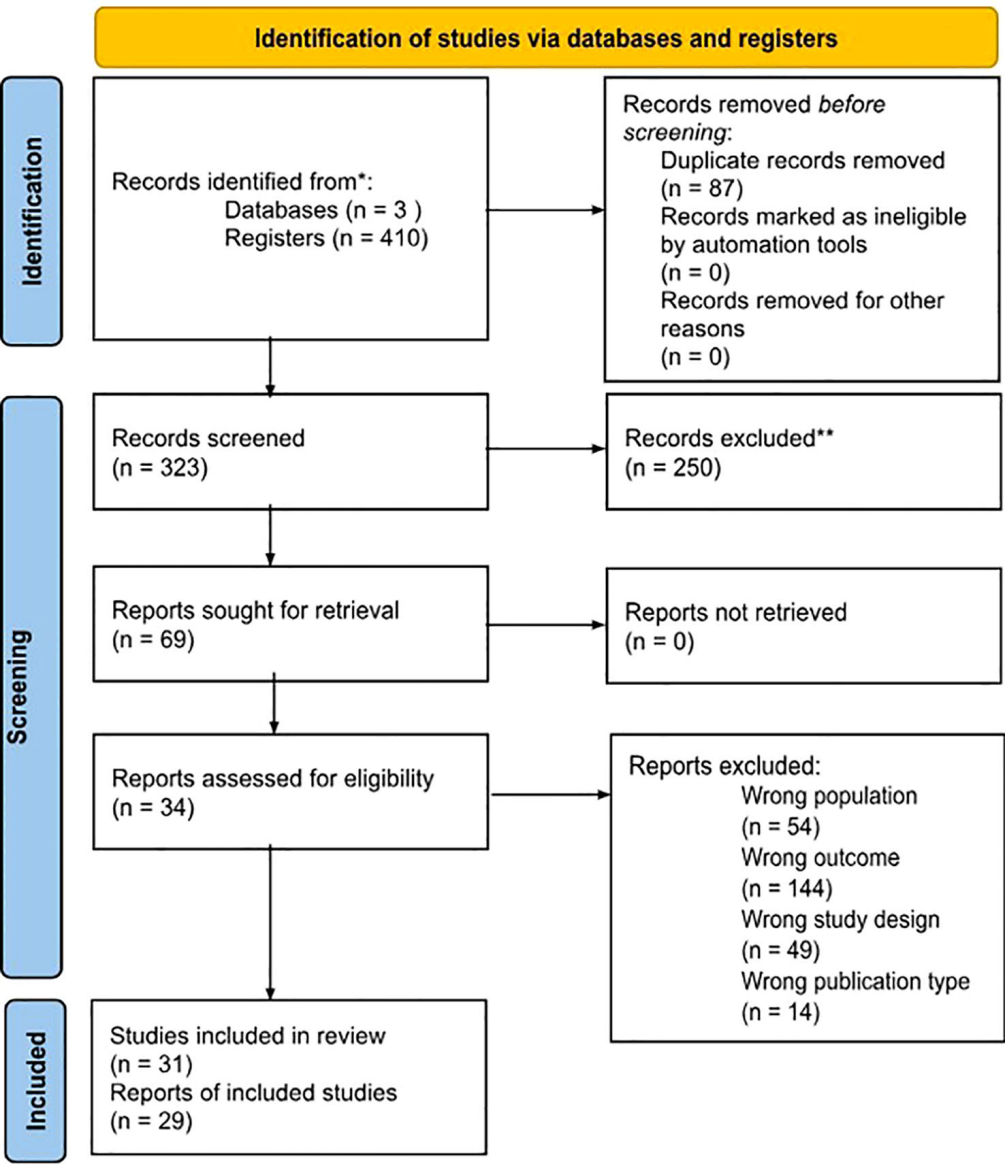
PRISMA flow diagram.

Initial searches conducted across PubMed, Web of Science, and Scopus yielded 410 records, which were subsequently reduced to 323 after removing duplicates and excluding 19 background articles and 14 book chapters. The PICO framework (Population, Intervention, Comparison, Outcome) guided the screening process by identifying Population with ASD (age and gender information), interventions focused on sensory stimulation (hypersensitivity evaluation), comparisons between baseline and post-intervention in ASD and TD groups (initial and final evaluation), and Outcomes related to responses to lighting and acoustics (lighting and acoustics). Applying PICO criteria, 144 studies were excluded for incorrect outcomes, 54 for inappropriate populations, and 49 for incompatible designs, resulting in 29 studies selected for in-depth analysis. The final 29 studies that met all eligibility criteria were included in the qualitative synthesis, reflecting sensory research trends in ASD. A PRISMA flow diagram summarized the selection process (**Figure 1**).

### 2.5 Evaluation quality

A 15-question questionnaire, derived from the DISCERN tool (11), was utilized to assess the quality of the studies. The instrument is based on 16 questions: Q1. Are the aims clear? Q2. Does it achieve its aims? Q3. Is it relevant? Q4. Is it clear what sources of information were used to compile the publication (other than the author or producer)? Q5. Is it clear when the information used or reported in the publication was produced? Q6. Is it balanced and unbiased? Q7. Does it provide details of additional sources of support and information? Q8. Does it refer to areas of uncertainty? Q9. Does it describe how each treatment works? Q10. Does it describe the benefits of each treatment? Q11: Does it describe the risks of each treatment? Q12. Does it describe what would happen if no treatment is used? Q13. Does it describe how the treatment choices affect overall quality of life? Q14. Is it clear that there may be more than one possible treatment choice? Q15. Does it provide support for shared decision-making?

The DISCERN tool indicated a moderate overall research quality, as studies consistently defined aims, employed appropriate methods, and provided transparent, evidence-based conclusions. While DISCERN was initially developed for assessing consumer health information, it was selected here because many ASD hypersensitivity studies examine consumable sensory materials, technologies, or therapeutic environments. Although minor methodological challenges were noted, such as small sample sizes and insufficient blinding details, they did not undermine the robustness of the findings. The consistency across high-quality studies, coupled with supplementary research from large-scale surveys and underrepresented groups, reinforces sensory hypersensitivity as a fundamental characteristic of ASD, characterized by atypical sensory adaptation, modality-specific hypersensitivities, and variations in context processing.

Out of the 29 articles reviewed, 28 are rated as Moderate quality, while Jertberg et al. (12) stands out as the sole exception, achieving High quality. This distinction arises from the DISCERN instrument’s penalization of basic research when assessed as “consumer/patient information.” Structurally, articles demonstrate strong performance (scores of 1-2) in objectives, scholarly relevance, and source citation, indicating technical soundness. However, a notable decline occurs in Q13-Q15, where scores drop to Low/Very Low. These areas, which address the daily quality of life (Q13), provide treatment options (Q14), and support shared decision-making (Q15), are often overlooked, as many studies prioritize mechanisms over practical applications. Jertberg et al. (12) scores “High” due to its diverse sample, including adults with over 60% women, and its ecological context, which mitigates bias. Conversely, articles like those by Chien (13), Foster (14), Green (4), and Ahlfors (15) score “Very Low” due to biased samples (primarily young men/boys) and artificial contexts, which lower their overall averages (**Table 1**).

**Table 1:** The DISCERN tool to measure research quality.

No.	Q1	Q2	Q3	Q4	Q5	Q6	Q7	Q8	Q9	Q10	Q11	Q12	Q13	Q14	Q15	Q16	Quality score
1	1	2	2	1	1	2	3	2	2	3	4	4	2	4	5	1	High
2	1	2	2	1	1	5	3	4	2	3	4	4	5	4	5	3	Moderate
3	1	2	2	1	1	5	3	4	2	3	4	4	5	4	5	3	Moderate
4	1	2	2	1	1	4	3	4	2	3	4	4	2	4	5	2	Moderate
5	1	2	2	1	1	4	3	4	2	3	4	4	5	4	5	3	Moderate
6	1	2	2	1	1	4	3	4	2	3	4	4	5	4	5	3	Moderate
7	1	2	2	1	1	3	3	4	2	3	4	4	5	4	5	3	Moderate
8	1	2	2	1	1	4	3	4	2	3	4	4	5	4	5	3	Moderate
9	1	2	2	1	1	4	3	4	2	3	4	4	5	4	5	3	Moderate
10	1	2	2	1	1	3	3	4	2	3	4	4	5	4	5	3	Moderate
11	1	2	2	1	1	4	3	4	2	3	4	4	5	4	5	3	Moderate
12	1	2	2	1	1	4	3	4	2	3	4	4	5	4	5	3	Moderate
13	1	2	2	1	1	4	3	4	2	3	4	4	5	4	5	3	Moderate
14	1	2	2	1	1	5	3	4	2	3	4	4	5	4	5	3	Moderate
15	1	2	2	1	1	5	3	4	2	3	4	4	5	4	5	3	Moderate
16	1	2	2	1	1	5	3	4	2	3	4	4	5	4	5	3	Moderate
17	1	2	2	1	1	4	3	4	2	3	4	4	5	4	5	3	Moderate
18	1	2	2	1	1	4	3	4	2	3	4	4	5	4	5	3	Moderate
19	1	2	2	1	1	4	3	4	2	3	4	4	5	4	5	3	Moderate
20	1	2	2	1	1	3	3	4	2	3	4	4	5	4	5	3	Moderate
21	1	2	2	1	1	4	3	4	2	3	4	4	5	4	5	3	Moderate
22	1	2	2	1	1	4	3	4	2	3	4	4	5	4	5	3	Moderate
23	1	2	2	1	1	5	3	4	2	3	4	4	5	4	5	3	Moderate
24	1	2	2	1	1	3	3	4	2	3	4	4	5	4	5	3	Moderate
25	1	2	2	1	1	4	3	4	2	3	4	4	5	4	5	3	Moderate
26	1	2	2	1	1	5	3	4	2	3	4	4	5	4	5	3	Moderate
27	1	2	2	1	1	4	3	4	2	3	4	4	5	4	5	3	Moderate
28	1	2	2	1	1	5	3	4	2	3	4	4	5	4	5	3	Moderate
29	1	2	2	1	1	4	3	4	2	3	4	4	5	4	5	1	Moderate

Based on the answers to all of the above questions, rate the overall quality of the publication as a source of information about data treatment choices:

Low 4-5 (serious or extensive shortcomings)

Moderate 3 (potentially important but not serious shortcomings)

High 1-2 (minimal shortcomings)

## 3 Results

Research findings reveal a significant imbalance in sensory research, with auditory paradigms dominating literature. Of the 29 included studies, 16 focused on auditory stimulation, while only 2 exclusively examined visual stimulation, and 11 investigated audiovisual paradigms. Participant ages ranged from 2 to 75 years, encompassing both ASD and TD groups. Sensory paradigms included steady-state auditory responses, event-related potentials, sensory gating, habituation, multisensory integration, and startle reflex measures. This trend reflects the accessibility and established neural markers of auditory research, whereas visual hypersensitivity, such as lighting sensitivity, remains comparatively underexplored (4, 16).

### 3.1 Summary of sensory outcomes

Data extracted from the studies encompassed key elements such as author and publication year, participant characteristics, the type of sensory stimulation employed, sensory functional variables, and sensory well-being variables. Due to substantial heterogeneity in outcomes, methodologies, and measures, a descriptive synthesis was conducted instead of a meta-analysis (**Table 2**), which includes author/year, participant demographics, stimulation type, functional sensory variables, well-being variables, and main conclusions. Broad patterns observed across the studies include:

**Table 2:** Summary of sensory outcomes.

Author	Population age	Population gender M/F	Stimulation	Functional sensory variable	Well-being sensory variable	Results/Conclusion
Jertberg et al, 2024 (12)	44.9 (14.0) ASD 33.1 (13.0) TD (496 ASD and 373 TD)	64.5% ASD-F 51.2% TD-F	audio-visual (1000–2000 ms)	speech perception		Differences in multisensory perception persist and evolve in autistic individuals as they age. While sensory perception often declines over time, multisensory integration can help maintain balance.
Ahlfors et al, 2024 (15)	13.6 (0.6) 13.1 (0.6) (22 ASD and 31 TD)	18.1% ASD-F 12.9% TD-F	auditory (25–80 Hz)	steady-state response (ASSR)		The Auditory Steady-State Response (ASSR) shows no significant differences between TD and ASD individuals across various stimulation frequencies, suggesting that neural abnormalities in ASD vary across sensory domains.
Cary et al, 2024 (17)	12.53 (2.53) ASD 12.81 (2.63) TD (13 ASD and 13 TD)	15% ASD-F 53% TD-F	auditory (0.25 − 8 Hz)	sensory overresponsivity		Autistic children may experience reduced habituation to auditory stimuli, with neural indicators connected to their traits. Providing sensory accommodations, like sensory-motor activities and structured environments, can help manage sensory over responsivity.
Jamal et al, 2024 (22)	9.8 ASD and 11.2 TD (10 ASD and 10 TD)	20% ASD-F 60% TD-F	auditory (250 Hz to 8,000 Hz)	Auditory sensory gating		The most effective sound for suppression was waterfall, followed by rainfall, white noise, and Quranic recitation. Apart from white noise, commonly used in clinics, other sounds like waterfall, rainfall, and Quranic recitation may be more soothing and comfortable, especially for individuals with sensory processing challenges, such as children with ASD.
Goris et al, 2022 (30)	35.63 (0.83) ASD 7.54 (0.81) TD (27 ASD and 27 TD)	29.6% ASD-F 29.6% TD-F	auditory		Context sensitive	Individuals with autism may not be as influenced by first impressions as TD individuals. This suggests they update their models more quickly or let go of older information.
Crosse et al, 2022 (29)	21.0 (8.66) ASD 22.5 (9.53) TD (139 ASD and 225 TD)	24% ASD-F 51% TD-F	auditory (1 kHz), visual (red disk) and audiovisual (disk/tone)	switching sensory modality		Teenagers with ASD show less difficulty when switching from auditory to visual stimuli, possibly due to changes in cross-sensory inhibition or reduced reliance on short-term statistical cues.
Dwyer et al, 2021 (40)	38.5 (6.02) ASD 37.1 (6.46) TD (130 ASD and 81 TD)	15% ASD-F 36% TD-F	auditory	sensory event-related potential		Reduced response amplitudes in ASD were noted, but significant overlap between diagnostic groups limited the detection of meaningful differences. This overlap, along with varied neural response patterns, highlights the role of inter-individual variability in neurotypes such as ASD and TD.
Ainsworth et al, 2021 (38)	15.4 (4.8) ASD 15.4 (5.8) TD (45 ASD and 111 TD)		auditory (3500 Hz), visual (flash) and audiovisual (tone and flash)	multisensory integration		Neurotypical individuals showed higher redundancy gain (RG) than autistic individuals, with efficient multisensory integration (MSI) breaking the race model, especially in older participants. Autistic groups had less MSI facilitation and minimal race model violations.
Osório et al, 2021 (9)	5.40 (2.61) ASD 6.40 (2.84) TD (168 ASD and 439 TD)	15% ASD-F 47% TD-F			sex differences in sensory processing	The magnitude of differences in sensory processing between males and females is more pronounced in ASD children compared to TD children. Females tend to experience more severe symptoms, particularly in Hearing, as well as Balance and Motion subscales.
Kawakami et al, 2020 (25)	28.13 (7.16) ASD 29.00 (10.39) TD (15 ASD and 18 TD)	40% ASD-F 39% TD-F	auditory (1 kHz), visual (red disk) and audiovisual (disk/tone)	multisensory integration		Individuals with ASD or higher levels of autistic traits were less affected by sound-induced flash illusion (SIFI) than the TD group, indicating that their visual perception is less influenced by auditory input and highlighting potential difficulties in integrating audio-visual information.
van Laarhoven et al, 2020 (31)	18.64 (2.11) ASD 18.93 (1.22) TD (29 ASD and 29 TD)	28% ASD-F 21% TD-F	auditory (25–80 Hz)	hypo- and hyperresponsiveness		Autistic individuals process visual and auditory information in unique ways. For instance, when sound is unexpectedly absent in a sequence of videos where visual movement usually predicts the sound, autistic individuals show a stronger prediction error response.
Seymour et al, 2020 (39)	16.67 (3.2) ASD 16.69 (2.8) TD (18 ASD and 18 TD)	22% ASD-F 17% TD-F	auditory (0.45 − 8 Hz)	Multisensory Integration		The ASD group had slower visual and auditory processing and delayed Multisensory Integration (MSI) onset. Early MSI deficits in ASD may be balanced by later attention, leading to normal MSI behavior.
Donkers et al, 2019 (34)	7.6 (2.2) ASD 7.0 (2.0) TD (28 ASD and 39 TD)	21% ASD-F 23% ASD-F	auditory (250 Hz to 8,000 Hz)	sensory and attentional processing		The connection between Hyporesponsive and Sensory Seeking behaviors in ASD and sensory and attentional neural processes is influenced by both bottom-up and top-down mechanisms. Early detection and intervention are essential to prevent neural abnormalities, support development, and address core ASD features.
Chien et al, 2019 (13)	20.6 (4.1) ASD 20.4 (3.1) TD (34 ASD and 34 TD)	5.9% ASD-F 5.9% TD-F	auditory	Sensory gating		Adolescents and young adults with ASD had normal P50 and P200 responses but showed N100 gating deficits. The larger N100 amplitude in response to S2 is significant because it relates to daily hypersensitivity and could be used as a marker for hypersensitivity, regardless of an ASD diagnosis.
Green et al, 2019 (4)	13.28 (3.35) ASD 13.53 (2.79) TD (42 ASD and 27 TD)	9.5% ASD-F 30.6% TD-F	auditory (1 kHz), visual (red disk) and audiovisual (disk/tone)	Sensory overresponsivity		Children with autism experience sensory stimuli in unique ways. Some find it hard to adjust to repeated stimuli, while others show distinct brain activity patterns. These differences emphasize how regulatory mechanisms influence behavior.
Keith et al, 2019 (23)	14.2 (1.4) ASD 14.8 (1.2) TD (25 ASD and 21 TD)	8% ASD-F 9.5% TD-F	auditory	hyperresponsivity to noise		Noise affects task performance differently based on difficulty levels. While harder tasks may not show obvious performance impacts, individuals with ASD experience significant underlying physiological effects. These effects can influence behavior and health, highlighting the need to account for sensory and task demands in this population.
Millin et al, 2018 (19)	(24 ASD and 29 TD)		auditory (3500 Hz), visual (flash) and audiovisual (tone and flash)		Adaptation	The study found that individuals with ASD had a stronger and more sustained response in the auditory cortex, but not the visual cortex, compared to neurotypical controls during fixed-interval timing.
Hudac et al, 2018 (18)	12.29 (3.56) ASD 13.27 (2.34) TD (108 ASD and 34 TD)	32% ASD-F 24% TD-F	auditory (sinusoid stimuli of either 1000 or 750 Hz)	Atypical electrophysiological responses		ASD individuals had a stronger reaction to new sounds (P3a response). Their brain responses changed more slowly (N1 and P3a). These differences were linked to sensory-seeking behaviors, not general cognition. They showed heightened responses to frequency differences and slower habituation to novel sounds.
Vlaskamp et al, 2017 (20)	11.1 (1.4) ASD 10.9 (1.3) (35 ASD and 38 TD)	20% ASD-F 29% TD-F	auditory (1000 Hz frequency and intensity of 75 dB)	event-related potentials		Reduced mismatch negativity in children with ASD suggests that children with ASD may be less responsive to environmentally deviant stimuli at an early (sensory) level. P3a-amplitude was increased in ASD, implying a hyper-responsivity at the attentional level.
Remington & Farnie, 2017 (21)	23.8 (4.0) ASD 26.8 (2.5) TD (20 ASD and 20 TD)	37% ASD-F 31% TD-F	auditory (250 to 8000 Hz)	increased perceptual capacity		Autistic individuals often describe their hearing as being “like microphones,” picking up all the sounds around them. This reflects an enhanced ability to process sounds rather than a lack of focus. To help reduce distractions, minimizing background noise and increasing task complexity can help focus attention.
Bao et al, 2017 (26)	18.75 (4.74) ASD 18.95 (5.06) (20 ASD and 20 TD)	20% ASD-F 10% TD-F	Audio-visual (3500 Hz beep and 12.5 ms flash)	flash-beep sensory processing		The fission illusion is equally strong in individuals with ASD and TD individuals, but the fusion illusion is more noticeable in the ASD group. This is likely because the auditory system is better at judging temporal aspects of the task, making the auditory stimulus more dominant.
Takahashi et al, 2017 (35)	15.6 (5.6) ASD 15.8 (4.4) TD (12 ASD and 24 TD)	17% ASD-F 37% TD-F	Auditory (65–105 dB, increments in five intensities)		Pulse stimuli and auditory hyper-reactivity	Children with ASD have a longer acoustic startle response (PSR) and stronger reaction (ASR) at 85 dB. They also show a longer peak startle latency (PSL) and stronger response to softer sounds. These are consistent neurophysiological markers of ASD. Prepulse inhibition (PPI) with a 70 dB prepulse may serve as a marker for psychiatric traits beyond ASD in children.
Noel, 2017 (28)	12.3 (3.05) ASD 11.6 (3.79) TD (26 ASD and 26 TD)	7.6% ASD-F 50% TD-F	audiovisual (3500 Hz 10 ms)		temporal recalibration	In individuals with ASD, the temporal binding window (TBW), or the precision of audiovisual timing, is most affected by speech stimuli compared to simple and complex non-speech stimuli.
Skewes et al, 2016 (32)	27.44 (0.79) ASD 25.79 (0.43) TD (16 ASD and 19 TD)	31% ASD-F 10.5% TD-F	auditory (100–800 Hz)		difficulties localizing sounds in space	The challenges individuals with ASD face in processing auditory location cues can affect their sensory experiences, social interactions, and communication.
Stewart et al, 2016 (37)	13.1 (2.8) ASD 13.6 (2.7) TD (25 ASD and 33 TD)		auditory (1,600-4,000 Hz) Visual (2,000 ms)	bisensory facilitation		High-functioning children with ASD often exhibit atypical sensory behaviors, especially in the auditory domain. This may be linked to auditory sensitivities and challenges with auditory filtering, which are recognized as some of the earliest ASD symptoms in retrospective videotape studies. However, bisensory facilitation of simple non-verbal stimuli may not be impaired in ASD.
Foster et al, 2016 (14)	12.4 (2.4) ASD 12.9 (2.5) TD (35 ASD and 40 TD)	8.5% ASD-F 62.5% TD-F	auditory (269.7 Hz to 813.3 Hz)	global and local processing		Tasks with simple demands may improve perceptual performance in ASD by requiring less neural complexity. Complex tasks may show impaired performance due to higher neural demands. The divided attention task, which monitors auditory patterns over time, likely requires more working memory and simultaneous processing of global and local levels.
Turi et al, 2016 (27)	29.2 (5.2) ASD 27.1 (2.83) TD (16 ASD and 16 TD)	25% ASD-F 18.7% TD-F	visual and auditory (512 ms)		adaptation	Autistic adults adapt slower to audiovisual synchrony than typical adults, who show strong adaptation. Additionally, in typical adults, adaptation strength correlates with the perceived audiovisual synchrony window, but this is not observed in the autistic group.
Daluwatte et al, 2016 (41)	10.7 (3.4) ASD 10.9 (2.9) TD (152 ASD and 107 TD)	11% ASD-F 26% TD-F	visual (100 ms green light flash)	pupillary light reflex		Children with ASD often have significant sensory dysfunction, which is linked to decreased pupillary light reflex constriction amplitude. This dysfunction may be related to autonomic nervous system issues.
Chen et al, 2016 (24)	11.0 (1.3) ASD 10.9 (1.1) TD (16 ASD and 16 TD)		visual (eyes open and closed) and tactile (light touch and no touch)	light fingertip touch		A light fingertip touch helps reduce sway more in children with ASD than in TD. This suggests it could be a useful tool to improve balance in children with ASD

ASD, Autism syndrome disorder.

ASSR, Auditory Steady-State Response.

TD, Typical development.

M, Male.

F, Female.

MSI, Multisensory Integration.

### 3.2 Auditory and visual hypersensitivity

Auditory and visual hypersensitivity in ASD significantly differ from typical sensory processing, reflecting unique neural and perceptual mechanisms as summarized in **Table 2**. In the Auditory Hypersensitivity domain, individuals with ASD exhibit reduced habituation and adaptation compared to typically developing peers, characterized by prolonged neuronal activity in response to repetitive stimuli (17–19). These processing variances are further complicated by sensory gating deficits that impair the filtering of irrelevant sounds, evidenced by higher N100 amplitudes associated with hypersensitivity (13) and increased responsiveness to distracting stimuli (20). Paradoxically, this profile may reflect an enhanced perceptual capacity akin to a “microphone effect,” allowing for the simultaneous processing of multiple auditory sources (21). Furthermore, physiological responses are highly dependent on the nature of the stimulus; while task-irrelevant noise can have a negative impact, natural sounds such as rain are often perceived as calming (22, 23).

In relation to visual hypersensitivity (**Table 2**), physiological evidence suggests pupillary autonomic dysfunction, where altered pupillary light reflexes indicate a biological mechanism underlying photophobia (24). Concurrently, perceptual processing in ASD is characterized by a strong detail-oriented bias; individuals tend to prioritize specific visual elements over the global context, a processing style that renders them less susceptible to visual illusions requiring global integration (25, 26).

In the domain of audiovisual integration (**Table 2**), deficits are marked by a slower adaptation to audiovisual asynchrony (27). Furthermore, individuals with ASD encounter significant challenges in merging auditory and visual inputs, a difficulty attributed to a wider temporal integration window that disrupts the synchronization necessary for cohesive multisensory perception (12, 28).

### 3.3 Age-related findings

Age significantly influences sensory hypersensitivity in autism, as evidenced by research indicating persistent sensory differences in both adolescents and adults (12, 19). While sensory acuity may decline with age, autistic adults often retain distinctive sensory responses, such as improved multisensory integration (29). Longitudinal studies suggest gradual coping improvements in sensory input during adolescence, but cross-sectional data show hypersensitivities can persist into later life. Middle-aged and older adults exhibit stable sensory sensitivities, challenging assumptions of age-related decline (14). These findings underscore the need to consider all age groups in sensory research. Age bias in the studies is evident in the focus on childhood and adolescence, with a limited representation of middle-aged and older adults. ASD participants have an average age of 18.34 years (SD 9.67), but this wide range (5.4 to 44.9 years) reveals a bias in their age distribution. Few studies provide valuable insights into the persistence of multisensory perception in older adults with ASD by examining a middle-aged cohort (mean age 44.9 years) (12). Furthermore, several studies have indicated significant age differences between ASD and TD groups (30). This could lead to confounding results, as it may attribute differences in sensory processing to aging rather than to autism.

### 3.4 Gender-related findings

The analysis of gender distribution reveals one of the most significant and problematic biases in current ASD research, with women comprising only 21.79% of participants on average, compared to 31.42% in typically developing (TD) control groups. This disparity, which reflects a historical diagnostic bias, severely limits the generalizability of findings to autistic women. There are several studies that illustrate this problem, including Chien et al. (13) with 5.9% and Foster et al. (14) with 8.5%. The low female participation observed should be interpreted with caution, as the underlying mechanisms may differ from those experienced by women with autism spectrum disorders. These women may have distinct sensory profiles or coping strategies compared to women without autism spectrum disorders. Most studies reviewed reported a significant gender imbalance, with approximately 80% of participants being male. This underrepresentation of autistic females is a critical gap, as emerging evidence suggests distinct sensory coping strategies, hormonal influences, and masking tendencies across genders. While few studies explicitly examined sensory hypersensitivity differences between autistic males and females, findings were inconclusive due to limited female representation. Indirect observations suggest autistic females may experience hypersensitivities as intensely as males, if not more so. Recent investigations outside the initial sample indicate autistic individuals assigned female at birth tend to have heightened overall sensory sensitivities, reinforcing the need for more gender-balanced research.

### 3.5 Context related findings

Individuals with ASD exhibit atypical prediction error responses in audiovisual omissions (31). Notably, some research suggests enhanced updating of sensory models rather than rigidity (30). The functional impact of these differences varies, with certain sensory differences associated with behavioral distress and others linked to enhanced perceptual abilities. Further EEG research employing multi-timescale oddball paradigms indicates that autistic adults can update their sensory models more rapidly, exhibiting a reduced “primacy bias” and diminished influence of initial context on mismatch negativity. This suggests a faster weighting of novel sensory information (30). These findings show that people with ASD focus on immediate details rather than context when processing sensory information. Additionally, autistic participants demonstrate atypical auditory filtering and sensory gating, such as reduced efferent suppression of otoacoustic emissions and differential effects of masking sounds. These factors can make subtle changes in background noise or sound texture disproportionately influential on comfort and behavior (22). The impact of sensory processing differences becomes more pronounced as contextual complexity increases, with subtle changes in lighting or sound significantly influencing behavior. These insights underscore the significance of creating autism-friendly environments to mitigate sensory overload.

### 3.6 The mechanisms, methodological gaps, and ecological validity

**Figure 2:**
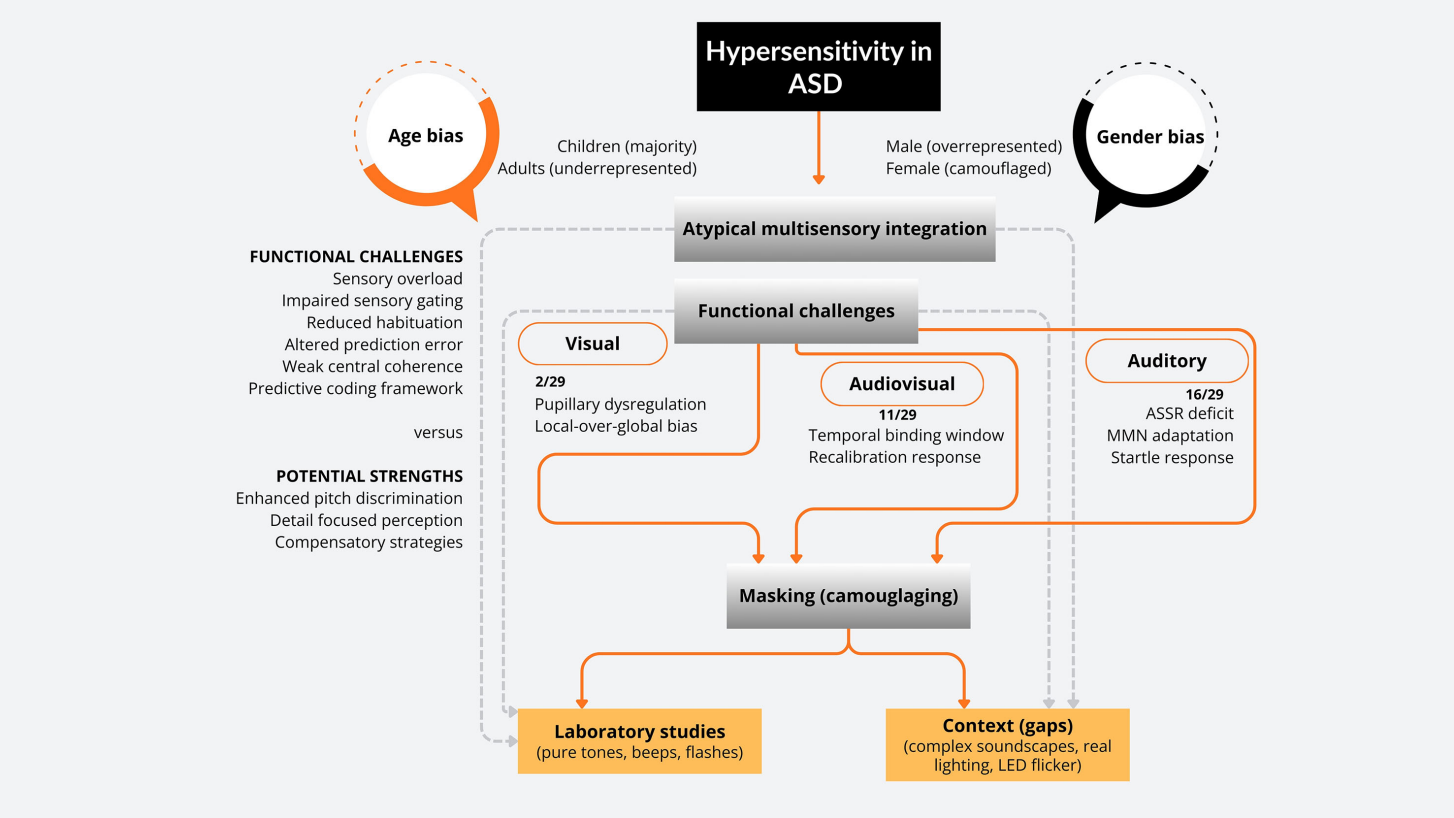
Neurophysiological mechanisms and methodological gaps in ASD studies.

A conceptual model (**Figure 2**) illustrates how neurophysiological mechanisms interact with methodological biases found in recent literature. In this model, sensory hypersensitivity in ASD is understood as the result of disrupted homeostasis in the processing of stimuli. As part of hypersensitivity, enhanced perceptual functioning (EPF) is one of the main challenges, allowing superior auditory pitch and visual detail discrimination. Due to predictive coding frameworks and weak central coherence (WCC), audiovisual omission paradigms exhibit altered anticipatory processing and enlarged prediction errors.

Over-responsiveness is also associated with reduced habituation and impaired sensory gating. Repeated sounds are amplified, mismatch negativity adaptation is reduced across blocks, and P50 suppression ratios are deficient, indicating redundant sensory input is not filtered. However, these mechanisms are not independent; they are modulated by crucial variables like gender and age. Despite recurring temporal binding window abnormalities, developmental trajectories suggest that multisensory integration deficits observed in childhood may engage compensatory processes in adulthood. Females, on the other hand, are often able to internalize discomfort, resulting in underdiagnosis and exclusion from experimental studies focused on visible reactivity.

The study findings incorporate a dichotomy between controlled laboratory and ecological context. In laboratory studies, pure tones (500–1000 Hz), brief visual flashes, and clinical white noise dominate stimuli. Unlike laboratory paradigms, real-world soundscapes and lighting are complex and unpredictable (traffic, classroom chatter, LED flicker). Ony one study examined naturalistic auditory stimuli, such as waterfalls and rainfall, which are more effective in regulating physiological responses (22). Despite clinical reports, no studies have evaluated modern LED lighting, photophobia, or flicker frequencies between 100–200 Hz.

## 4 Discussion

Results indicated that most studies on sensory stimulation have concentrated on auditory versus lighting experiments. Out of the 29 studies, only two focused on visual stimuli, 11 were audio-visual, and 16 were auditory. Studies have demonstrated that individuals with ASD may encounter difficulties in processing complex auditory stimuli, such as distinguishing the overall pitch structure in nine-tone melodies, particularly under conditions of heightened sensory input or increased attention demands. These challenges emphasize the need for more comprehensive research into the way autistic individuals process sensory information and adapt to environments characterized by varying levels of sensory stimuli.

In **Figure 2**, the conceptual model illustrates the interaction between neurophysiological mechanisms and methodological biases identified in recent literature. According to this model, sensory hypersensitivity in ASD is the result of disturbed homeostasis in stimulus processing and not a simple enumeration of symptoms. Even though ASD sensory profiles appear to be heterogeneous, this may not be due to inherent neurobiological differences, but to systematic measurement errors. Male children’s research using oversimplified laboratory stimuli inevitably obscures the sensory realities of women, older adults, and individuals who navigate complex environments on a daily basis.

A core component of this model lies in the mechanisms of reduced habituation and impaired sensory gating, which are the primary determinants of overreacting to auditory and visual stimuli. The model emphasizes, however, that these mechanisms are not independent; rather, they are modulated by important and under-researched variables, such as age and gender. As for multisensory integration, developmental trajectory suggests that it may circumvent compensatory processes during adulthood, even when hypersensitivity is present. Identifying a gender bias reveals a gap in understanding ‘sensory camouflaging’ strategies in females, which lead to significant clinical differences in research findings. Five primary topics warrant discussion in relation to sensory overstimulation and light/sound hypersensitivity: gender-biased studies, age-biased studies, context bias, local-global stimuli bias, and the adaptive bias.

### 4.1 Gender bias

The reviewed studies exhibit a notable underrepresentation of women, highlighting a significant gap in the field of biomedical research. The majority of “findings about autism” pertain to autism in men. The issue extends beyond equity to scientific accuracy. A personalized and effective intervention cannot be developed if we disregard potential biological and social differences in sensory processing (such as masking, which is more prevalent in women). The bias in most autism research stems from its predominant inclusion of males, reflecting the fact that males account for 80% of ASD diagnoses. The gender imbalance in study populations presents a significant gap in comprehending the phenotypic similarities or differences between males and females with ASD. For instance, while no performance disparities were observed between males and females in TD groups, it cannot be assumed that these findings extend to females with ASD. The absence of female representation in studies restricts insights into potential gender-specific sensory and perceptual variations, such as differences in auditory and hierarchical perception. Future research must address this gap by including diverse gender groups to better understand the sensory impact and behavioral responses in both males and females with ASD (9).

The reviewed studies predominantly included male participants, revealing a gender imbalance in the evidence. Few studies directly compared sensory hypersensitivity between autistic males and females, with findings generally failing to distinguish between the sexes due to limited female representation. Indirect observations suggest autistic females may experience hypersensitivities as intensely as males, if not more. However, conclusive comparisons are rare due to sample biases. These findings underscore the need for more gender-balanced research. Emerging observations indicate that autistic individuals assigned female at birth often report heightened sensory sensitivities, such as lower tolerance to intense stimuli, compared to males. This highlights the potential influence of sex-related factors on sensory experiences in ASD and the importance of addressing gender bias in future research.

### 4.2 Age bias

In most cases, ASD research is “infantilized.” While the emphasis on children and adolescents makes sense from a developmental perspective, the focus leaves a large gap in understanding how sensory hypersensitivity manifests, evolves, and impacts quality of life in adulthood and old age. Conclusions drawn from youth samples cannot, and should not, be generalized to the entire autistic population. A systematic and well-funded effort is needed to investigate autism across the lifespan (12) study. Age is a critical factor in understanding sensory adequacy to the environment in autism studies, as individuals with ASD may adapt to sensory stimuli over time. Research indicates that sensory responses, such as auditory and visual processing, can vary significantly across different age groups. For example, auditory global-local distinctions are less clear in ASD, particularly when considering age and attention effects. This highlights the importance of longitudinal studies that span from childhood through early adulthood, allowing researchers to observe developmental changes and adaptations. Such studies can provide insights into whether and how individuals with ASD adjust their sensory perceptions to environmental demands as they age (12, 14, 29).

Sensory hypersensitivity is a lifelong feature of autism, affecting individuals of all ages. For example, children and teenagers often react strongly to sensory stimuli like loud noises or bright lights. In middle-aged adults, sensory sensitivities persist, with some finding it difficult to cope with crowded or noisy environments. Among older adults, challenges such as filtering out background noise or adapting to sensory-rich settings are common. These experiences highlight that sensory sensitivities remain a consistent aspect of autism throughout life. Age plays a key role in sensory hypersensitivity outcomes. While basic sensory acuity may decline with age in both ASD and typical development, autistic adults often retain distinctive sensory responses. Older autistic individuals may show improved multisensory integration compared to younger ones, suggesting adaptation or life experience enhances their ability to combine inputs, such as sound and visual cues. However, evidence on age-related reduction in hypersensitivity is mixed: some studies suggest gradual coping improvements, while others indicate persistent hypersensitivities into later life.

### 4.3 Context bias

The majority of studies are conducted in highly controlled laboratory settings using simplified stimuli (pure tones, flashes of light). While this control is necessary for the isolation of neural mechanisms, it creates an ecological gap with the sensory reality of autistic people, who live in a multisensory, complex, and often chaotic environment. An auditory paradigm conducted in a soundproof room may have little relevance for understanding the distress experienced by an autistic person in a busy shopping mall. Temporal and contextual factors play a crucial role in neural information processing, particularly in understanding autistic traits and sensory behaviors. These factors influence how individuals with ASD perceive and respond to sensory stimuli, highlighting the need for studies that consider the dynamic interplay of time and context. For instance, while typically developed (TD) individuals tend to perceive “global” (whole) features before “local” (detailed) features in visual tasks, individuals with ASD often show a regional bias. This distinction may vary with age and attention, underscoring the importance of longitudinal studies that encompass a wide age range to capture developmental and contextual variations (30).

Autistic sensory responses depend on context, with studies suggesting that individuals with ASD process stimuli differently from neurotypical individuals. They may rely less on “first impression” cues or initial sensory information, instead updating their perceptions quickly. For instance, autistic teenagers showed less difficulty than controls when shifting attention from an auditory task to a visual one (29). This suggests that the usual cross-sensory interference or need for contextual adjustment might be reduced in ASD, possibly due to differences in how their brains inhibit competing sensory information or rely on short-term sensory predictions. Autistic individuals experience sensory processing differently, often focusing on immediate details rather than broader context. This can lead to sensory hypersensitivity, where environmental factors like lighting and sound significantly impact their responses. For example, soft, warm lighting promotes calmness, while harsh lighting or loud, unpredictable sounds can trigger discomfort or sensory overload. Autism-friendly environments, such as classrooms with soft lighting or quiet spaces with minimal distractions, are designed to reduce sensory challenges. In contrast, complex settings like crowded malls can worsen sensory overload. These differences highlight the need for tailored sensory environments to support autistic individuals.

### 4.4 Local stimuli versus global stimuli bias

The inherent difficulty autistic individuals face in processing multiple stimuli simultaneously may result in bias in their responses to local sensory stimuli. The cause of this is disruptions in neural information processing, which can result in overresponse or hyperreactivity of the sensory system. In contrast to individuals with TDs who tend to perceive global (whole) features before local (detailed) ones, individuals with ASDs tend to focus on particular details rather than the overall context (14). This distinction is particularly evident in auditory tasks, where global-local distinctions are less clear for ASD (4, 17). Autistic perception emphasizes local details over global patterns. Since ASD has difficulty applying sensory information to broader frameworks, it tends to focus on specific details rather than integration with wider input (32). They excel in identifying tonal variations but encounter difficulties in comprehending global auditory sounds. Specific sounds can also be remembered when detail-oriented approaches are employed.

When a visual flash is presented alongside a sound, autistic individuals may process the visual event independently, unlike neurotypicals. In visual tasks, they may focus on specific elements, like textures, but have difficulty understanding overall compositions. In social interactions, subtle cues may be noticed, but broader contexts are harder to comprehend. There is often a heightened sensitivity to sensory details among autistic individuals, such as subtle sounds or distinct patterns. As a result, it can be difficult for them to process complex scenes as a whole. This ASD characteristic, known as a local-over-global stimuli, affects perception by paying attention to details rather than the whole picture. Despite enhancing precision-based skills, working in everyday environments filled with sounds, patterns, and movement can be overwhelming. As a result of this perceptual imbalance, sensory overload is often reported in dynamic and busy settings.

### 4.5 Functional/adaptive versus well-being/comfort

Because of their heightened sensitivity, individuals on the autism spectrum often experience overstimulation from their living environments (2, 33, 34). Behavioral difficulties may result from sensory overresponsivity, such as covering their ears. However, most autism research focuses on the functional characteristics of ASD, such as sensory processing and perception, rather than addressing well-being and sensory comfort for autistic people (29, 34, 35). While enhanced perceptual abilities are sometimes viewed as a strength in autism (21, 32, 36), the effects of sensory overresponsivity go beyond functionality, affecting well-being and comfort in sensory environments. Autism is often associated with difficulties adjusting to ongoing stimuli. Research indicates that individuals with autism exhibit diminished habituation, characterized by heightened attentiveness to stimuli that others gradually become desensitized to (4, 17, 19, 35). Consequently, repeated auditory tones may elicit prolonged reactions, suggesting a slower or weaker neural adaptation process.

A study examining the Auditory Steady-State Response (a brain response to rhythmic sounds), revealed that individuals with autism exhibited comparable low-level neural tracking to controls, rather than a diminished response with repetition (15). However, other sensory adjustment measures indicated anomalies, suggesting that adaptive processes may vary across sensory domains or stimulus types in ASD (19). Sensory regulation is characterized by a slower response in ASD individuals. Consequently, their pupils exhibit heightened sensitivity to brightness and a prolonged adjustment period to mismatched visual and auditory stimuli, resulting in distinct physiological reactions to sensory input (24). Furthermore, their startle responses to loud sounds are more pronounced and persist longer, indicating a delayed sensory system recovery after being startled. A compromised sensory system can lead to persistent intense reactions. The difficulty in habituation persists throughout life, causing stress in sensory-rich environments. Fluctuating lights or humming sounds can be distressing due to the challenge in reducing their intensity, while gentle, consistent sensory input, such as soft background sounds, can serve as a calming and predictable anchor. Recognizing these sensory processing differences is crucial for managing sensory overload.

Autism involves complex alterations in sensory processing across different developmental stages, sensory modalities, and contexts. The basic integration of simple stimuli is intact in some studies (37), however, multisensory facilitation deficits persist with aging (38), as well as reduced neural synchronization to rhythmic inputs (39). Due to significant inter-individual variability in neural topography, it is difficult to identify uniform biomarkers for auditory processing in individuals with ASD (40). Compensatory mechanisms, such as the use of light tactile cues to correct postures in autistic children (41), demonstrate how sensory inputs can be harnessed to facilitate motor control in the face of processing difficulties.

Social adaptation and masking should be considered in the broader context of behavioral and neural findings, particularly in underrepresented groups. Studies on autistic females have shown that social coping strategies, such as camouflaging, may mask sensory difficulties, leading to underdiagnosis or delayed support (42). As a consequence, future research must move beyond simplistic paradigms and explicitly consider demographic factors such as gender and age in order to ensure that clinical and environmental guidelines reflect the diverse sensory realities of the entire autistic population.

## 5 Conclusions

The synthesis of 29 reviewed articles indicates that research on sensory hypersensitivity in autism has mainly focused on acoustic paradigms and controlled laboratory settings, while overlooking the impact of real-life environments, including housing, classrooms, offices, and public places. Despite significant developments, it is still unclear how modern lighting, for example LEDs with varying flicker frequencies or warm/cool tones, and complex soundscapes may affect fatigue, stress, and autistic behavior.

Moreover, long-term studies are lacking, particularly in adults and individuals over the age of 50, raising questions concerning how hypersensitivity develops with advancing age and whether age-related hearing loss mitigates or exacerbates the issues. Most existing research relies on simple sounds like “beeps” or pure tones, which fail to capture the complexities of real-world soundscapes. Furthermore, a gender bias complicates understanding, as studies tend to focus disproportionately on men, leaving uncertainty as to how autistic women process light and sound.

Several qualities have been highlighted by the revised articles, including an atypical adaptation to repeated stimulation, a reliance on immediate context, and a modality-specific hypersensitivity. Although these traits reflect broader challenges in integrating global information and adjusting to changing environments, they are also indicative of strengths in detail detection and perception precision. According to the review, age and gender bias undermine the validity of current research. Because women and older adults are underrepresented, most of what is “known” about autistic sensory processing applies only to a small percentage of the population. This issue has direct implications for clinical guidelines, diagnostic criteria, and the design of sensory environments that do not address the specific needs of women and adults.

There is also a dichotomy between controlled laboratory settings and ecological contexts. Future research must focus on gender-specific sensory gating and habituation analysis (2); ecological momentary assessment of hypersensitivity in real-world environments (workplaces, classrooms, public transit) (3); metabolic and cognitive costs of sensory camouflaging in females; and (4) translational studies linking neural biomarkers to concrete environmental changes. In addition to reversing historical inequities, this paradigm-shifting research can generate guidelines that benefit the entire autistic community. There is an urgent need to address these gaps as we move from wondering “What do we really know about sensory hypersensitivity in autism?” to “How little do we know about the experience of half the autistic population?”

## Data Availability

The original contributions presented in the study are included in the article/supplementary material, further inquiries can be directed to the corresponding author.
